# A Non-Linear Association of Triglyceride Glycemic Index With Cardiovascular and All-Cause Mortality Among Patients With Hypertension

**DOI:** 10.3389/fcvm.2021.778038

**Published:** 2022-01-27

**Authors:** Dan Zhou, Xiao-cong Liu, Lo Kenneth, Yu-qing Huang, Ying-qing Feng

**Affiliations:** ^1^Department of Cardiology, Guangdong Cardiovascular Institute, Guangdong Provincial People's Hospital, Guangdong Academy of Medical Sciences, Guangzhou, China; ^2^Department of Epidemiology, Centre for Global Cardio-Metabolic Health, Brown University, Providence, RI, United States; ^3^Department of Applied Biology and Chemical Technology, The Hong Kong Polytechnic University, Hong Kong, Hong Kong SAR, China

**Keywords:** insulin resistance, triglyceride-glucose index, hypertension, all-cause mortality, cardiovascular mortality

## Abstract

**Background:**

To investigate the association between insulin resistance (IR), quantified by triglyceride glycemic index (TyG index), cardiovascular mortality (CVM), and all-cause mortality (ACM) in hypertension patients.

**Methods:**

We included 8,554 patients with hypertension aged ≥18 years old from the 1999–2014 National Health and Nutrition Examination Surveys (NHANES). The status of CVM and ACM of participants were followed through December 31, 2015. Cox proportional hazards models and Kaplan-Meier survival curves were used to evaluate the relationship between TyG index, CVM, and ACM.

**Results:**

During a median of 82 months follow-up, 1,882 mortality cases had occurred, 434 of which were due to cardiovascular disease. The patients with hypertension with TyG ≥ 10 were older, had a higher chance of being smokers, were obese, had higher blood pressure, and had risk or had cardiovascular disease. In Cox proportional hazards models, compared with the patients with TyG <8, those with TyG ≥ 10 had 56% increased risk for ACM. On the other hand, no significant difference for CVM between the four groups were observed. In the restricted cubic spline regression models, the relationship between TyG index and ACM was non-linear. Subgroup analysis showed non-linear relationship between TyG index and ACM in elderly patients aged ≥60 years. The cut-off value of TyG for ACM was 9.45, and those with higher or lower than 9.45 had more risk of ACM. When TyG index was more than 9.52, the risk for CVM would increase among the whole group. Kaplan-Meier survival curves showed patients with TyG ≥ 10 had higher risk of ACM and CVM (Log rank *P* < 0.05).

**Conclusions:**

We demonstrated that the association between ACM and TyG index in elderly patients with hypertension aged ≥60 years was non-linear. However, TyG index was only more than 9.52, hence, the risk for CVM would increase among the whole hypertension group.

## Background

Hypertension is a primary risk factor for cardiovascular disease and all-cause mortality (ACM) (1, 2). Careful risk factor assessment plays an important role in hypertension patients. The metabolic effects of insulin resistance (IR), including hyperglycemia and dyslipidemia, appear to interact synergistically with increased blood pressure to cause vascular and kidney injury (3). IR is proposed to be important contributors to hypertension-caused organ damage, arterial stiffness (4), and left ventricular diastolic dysfunction (5) due to hypertension. Hence, higher IR is a risk factor for hypertension patients.

At present, Homeostatic model assessment IR (HOMA-IR), a method for assessing β-cell function and IR, is frequently applied, but its high cost and inconvenience make it difficult to use in large cohort study. Therefore, there has been no large cohort study examinate the prognosis value of IR in hypertension group.

Triglyceride-Glucose index (TyG index), first published by South American authors, showed a good correlation with the insulin clamp technique and HOMA-IR index. The TyG index, described to be a simple, convenient, and low-cost method, is of research interest in many countries in Asia and can be used to screen for IR in the Asian hypertensive community (6). Despite this, there is no evidence between TyG index and prognosis value in North America hypertensive community. Therefore, we conducted a retrospective cohort study to certify the relationship of cardiovascular mortality (CVM) and ACM in hypertension patients.

## Methods

### Study Population

**Figure 1:**
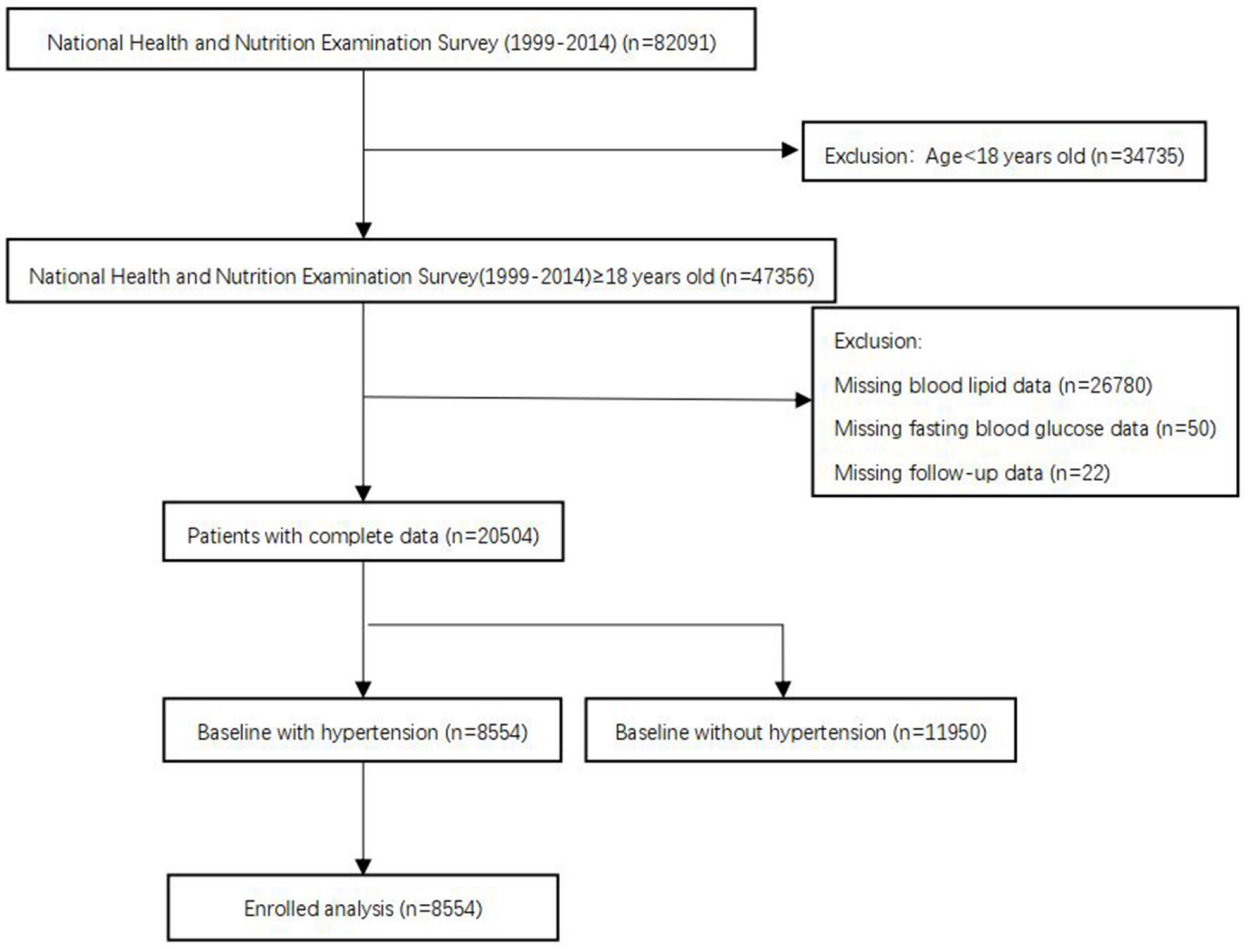
Study cohort.

The National Health and Nutrition Examination Surveys (NHANES), sponsored by the Centers for Disease Control and Prevention, is designed to assess the health status of US citizens. Data for analyses were taken from the NHANES 1999–2014 with a total of 82,091 participants. In our analysis, we included people aged ≥ 18 years old. Subjects without blood lipid data, fasting blood glucose data, follow-up data, or baseline without hypertension were excluded. After applying the criteria, we enrolled 8,554 participants for final analysis (Figure 1). The survival status of participants was followed up to December 31, 2015. The NHANES study protocol was approved by the Institutional Review Board of the Centers for Disease Control and Prevention. Informed consent was signed by all participants.

### Data Collection

Questionnaires were collected at baseline to acquire demographic information (age, gender, race, marital status, and education level), smoking status, personal medical history (cardiovascular diseases and diabetes), and medication history (antihypertensive drugs, hypoglycemic agents, lipid-lowering drug, and antiplatelet drugs).

Physical assessments were performed to examine height, weight, systolic blood pressure (SBP), and diastolic blood pressure (DBP). Blood samples were collected on an empty stomach in the morning and after 8 h. Total cholesterol (TC), triglyceride (TG), low-density lipoprotein cholesterol (LDL-C), high-density lipoprotein cholesterol (HDL-C), fasting blood glucose (FBG), and estimated glomerular filtration rate (eGFR) were also collected. Body mass index (BMI) was calculated using weight (kg) divided by the square of height (m^2^). Hypertension was defined as having an SBP ≥ 140 or/and DBP ≥ 90 mmHg, is confirmed to be taking antihypertensive medications, or has a self-reported history of hypertension (7). Diabetes was defined as having an FBG ≥ 126 mg/dl, having a self-reported hemoglobin A1c(HbA1C) ≥ 6.5%, or using hypoglycemic drug (8). TyG index was calculated by ln [fasting triglycerides (mg/dl) ^*^fasting glucose (mg/dl)/2] (9). eGFR was computed using Modification of Diet in Renal Disease (MDRD) formula (10).

### Clinical Outcome

All-cause mortality refer to death from any cause, cardiovascular disease, or cerebrovascular disease until December 31, 2015 were the primary outcomes. Mortality data were extracted from the 1999–2014 NHANES public-use linked mortality files. We examined the time from enrollment (date of interview) to mortality for censoring. The International Classification of Diseases, Tenth Revision codes (I00-I09, I11, I13, I20-I51) were used to define cardiovascular deaths. Any participant who was not matched with any death records was considered to be alive throughout the follow-up period.

### Statistical Analysis

Continuous variables were expressed as mean ± standard deviation for normally distributed variables or median (interquartile range) if the data were not normally distributed. Categorical variables were presented as number (n) and percentage (%). The one-way ANOVA, Kruskal–Wallis *H*-test or chi-square tests were used to assess differences according to baseline TyG index (TyG <8, 8 ≤ TyG <9, 9 ≤ TyG <10, TyG ≥ 10) in groups. Multivariable Cox regression analysis were used to estimate adjusted hazard ratio (HR) and 95% CI for mortality according to baseline TyG index in groups. Model I adjust for none. Model II adjust for age, gender, and race. Age, gender, race, smoking, marital status, education level, BMI, SBP, eGFR, TC, HDL-C, comorbidities (cardiovascular disease and diabetes), and medicine use (antihypertensive drugs, hypoglycemic agents, lipid-lowering drugs, and antiplatelet drugs) were included in Model III. We initially performed survival analysis using standardized Kaplan–Meier curves and Log rank test. The association between TyG index, ACM, and CVM was then examined by multivariate adjusted Cox restricted cubic spline regression models and used a generalized additive model to explore the non-linear relationship between TyG index and mortality. If non-linear relationships were identified, we used two-piecewise linear regression models to elucidate how the associations differed by the threshold point. The threshold value was estimated by trying all possible value and choosing the threshold point with highest likelihood. Finally, we conducted subgroup analyses, including age (<65 or ≥65 years), gender (male or female), race (white or non-white), and BMI (<25 or ≥25 kg/m^2^). *P* < 0.05 was considered statistically significant. R version 3.3.2 (R Foundation for Statistical Computing, Vienna, Austria) was used for all statistical analyses.

## Results

### Baseline Characteristics

The baseline characteristics of the analytical cohort according to TyG index in groups were showed in Table 1. In total, 8,554 patients (49.29% men) were included in this analysis with mean age of 60.12 ± 16.07 years. Of these, 4,309 (50.37%) participants were white, 4,632 (54.91%) married, 5,716 (67.70%) received high school or above education, and 4,178 (49.40%) never smoked. The patients with hypertension in group of TyG ≥ 10 were older, were more likely to be smokers, were obese, had higher blood pressure, and had higher risk or had cardiovascular disease. In addition, the proportion of participants with diabetes and cardiovascular disease was 30.33 and 19.36%, respectively. During a median follow up of 82 months, 1,882 cases of death have occurred, 434 of which were due to cardiovascular disease. All baseline variables differed significantly among the TyG index in groups (all *p* < 0.05).

**Table 1:** Demographic and clinical characteristics according to triglyceride-glucose index level.

	**Total**	**TyG <8**	**8 ≤T yG <9**	**9 ≤TyG <10**	**TyG ≥10**	***p***
Number	8,554	690	4,655	2,743	466	
Age, years	60.12 ± 16.07	54.13 ± 18.33	60.70 ± 16.32	60.92 ± 15.01	58.50 ± 13.96	<0.001
**Gender, n (%)**						<0.001
Male	4,216 (49.29)	327 (47.39)	2,203 (47.33)	1,412 (51.48)	274 (58.80)	
Female	4,338 (50.71)	363 (52.61)	2,452 (52.67)	1,331 (48.52)	192 (41.20)	
**Race, n (%)**						<0.001
Non-white	4,245 (49.63)	432 (62.61)	2,335 (50.16)	1,238 (45.13)	240 (51.50)	
White	4,309 (50.37)	258 (37.39)	2,320 (49.84)	1,505 (54.87)	226 (48.50)	
**Marital status, n (%)**						<0.001
Married	4,632 (54.91)	329 (48.53)	2,493 (54.31)	1,552 (57.29)	258 (56.33)	
Other	3,803 (45.09)	349 (51.47)	2,097 (45.69)	1,157 (42.71)	200 (43.67)	
**Education level, n (%)**						<0.001
Less than high school	2,727 (32.30)	173 (26.13)	1,412 (30.70)	945 (34.77)	197 (42.55)	
High school or above	5,716 (67.70)	489 (73.87)	3,188 (69.30)	1,773 (65.23)	266 (57.45)	
**Smoking, n (%)**						<0.001
No	4,178 (49.40)	365 (54.80)	2,366 (51.38)	1,251 (45.93)	196 (42.33)	
Yes	4,280 (50.60)	301 (45.20)	2,239 (48.62)	1,473 (54.07)	267 (57.67)	
Body mass index, kg/m^2^	30.31 ± 7.09	28.11 ± 7.30	29.76 ± 7.26	31.49 ± 6.51	32.15 ± 6.80	<0.001
Systolic blood pressure, mmHg	135.62 ± 21.35	133.21 ± 20.58	135.11 ± 21.62	136.53 ± 20.96	138.89 ± 21.55	<0.001
Diastolic blood pressure, mmHg	71.00 ± 16.07	71.07 ± 16.05	70.44 ± 16.21	71.79 ± 15.69	71.93 ± 16.69	0.004
eGFR, mg/min/1.73m^2^	79.31 ± 26.93	86.21 ± 27.81	79.29 ± 26.98	77.24 ± 25.85	81.55 ± 29.48	<0.001
Total cholesterol, mg/dL	197.68 ± 43.43	179.68 ± 39.45	193.14 ± 40.25	204.94 ± 43.47	226.94 ± 56.40	<0.001
HDL cholesterol, mg/dL	52.87 ± 16.31	66.46 ± 19.88	56.03 ± 15.75	46.36 ± 12.26	39.46 ± 11.65	<0.001
LDL cholesterol, mg/dL	115.31 ± 36.57	102.64 ± 32.30	116.05 ± 35.35	117.83 ± 38.74	109.09 ± 38.95	<0.001
Triglycerides, mg/dL	150.58 ± 117.66	52.23 ± 11.80	104.85 ± 28.96	203.81 ± 65.30	439.76 ± 291.68	<0.001
Fasting blood glucose, mg/dL	115.87 ± 42.86	92.76 ± 12.14	104.34 ± 18.47	125.30 ± 40.63	209.87 ± 94.41	<0.001
TyG index	8.86 ± 0.68	7.76 ± 0.21	8.56 ± 0.27	9.37 ± 0.26	10.51 ± 0.47	<0.001
**Comorbidities, n (%)**						
diabetes						<0.001
No	5,958 (69.67)	613 (88.84)	3,695 (79.39)	1,560 (56.89)	90 (19.31)	
Yes	2,594 (30.33)	77 (11.16)	959 (20.61)	1,182 (43.11)	376 (80.69)	
**Cardiovascular disease**						<0.001
No	6,821 (80.64)	570 (85.84)	3,776 (81.98)	2,124 (77.92)	351 (75.81)	
Yes	1,638 (19.36)	94 (14.16)	830 (18.02)	602 (22.08)	112 (24.19)	
**Treatment, n (%)**						
**Antihypertensive drugs**						<0.001
No	3,254 (38.04)	333 (48.26)	1,854 (39.83)	919 (33.50)	148 (31.76)	
Yes	5,300 (61.96)	357 (51.74)	2,801 (60.17)	1,824 (66.50)	318 (68.24)	
**Hypoglycemic agents**						<0.001
No	7,115 (83.18)	647 (93.77)	4,149 (89.13)	2,089 (76.16)	230 (49.36)	
Yes	1,439 (16.82)	43 (6.23)	506 (10.87)	654 (23.84)	236 (50.64)	
**Lipid-lowering drugs**						<0.001
No	6,309 (73.75)	569 (82.46)	3,498 (75.15)	1,936 (70.58)	306 (65.67)	
Yes	2,245 (26.25)	121 (17.54)	1,157 (24.85)	807 (29.42)	160 (34.33)	
**Antiplatelet drugs**						0.024
No	8,185 (95.69)	666 (96.52)	4,476 (96.15)	2,600 (94.79)	443 (95.06)	
Yes	369 (4.31)	24 (3.48)	179 (3.85)	143 (5.21)	23 (4.94)	
**Outcomes, n (%)**						
**Cardiovascular disease mortality**						<0.001
No	8,120 (94.93)	659 (95.51)	4,445 (95.49)	2,595 (94.60)	421 (90.34)	
Yes	434 (5.07)	31 (4.49)	210 (4.51)	148 (5.40)	45 (9.66)	
**All-cause mortality**						<0.001
No	6,672 (78.00)	582 (84.35)	3,650 (78.41)	2,125 (77.47)	315 (67.60)	
Yes	1,882 (22.00)	108 (15.65)	1,005 (21.59)	618 (22.53)	151 (32.40)	

*Q, quintiles; n, number; HDL, high density lipoprotein; LDL, low density lipoprotein; TyG, Triglyceride-glucose.*

*Values are mean ± standardized differences or n (%)*.

### Hazard Ratios (HRs) of TyG Index for ACM and CVM Risk

Table 2 reveals the estimated HRs and CIs of TyG index in relation to ACM and CVM. In the Model III, compared with the lowest group of TyG index (TyG <8), the HRs for ACM from other groups (8 ≤ TyG <9, 9 ≤ TyG <10, TyG ≥ 10) in the fully adjusted model were 0.90 (0.72 to −1.13, *p* = 0.36), 0.85 (0.66–1.10, *p* = 0.21), and 1.56 (1.14–2.15, *p* < 0.05), respectively (*P* for trend = 0.09).

**Table 2:** Multivariate Cox regression analysis of triglyceride-glucose index with cause-specific mortality.

	**Event rate/1,000 person-years**	**Model I**	**Model II**	**Model III**
		**HR (95%CI), *p* value**	**HR (95%CI), *p* value**	**HR (95%CI), *p* value**
**All-cause mortality**				
**TyG index group**				
TyG <8	24.07	1.0	1.0	1.0
8 ≤ TyG <9	29.16	1.19 (0.98, 1.45), 0.0841	0.84 (0.69, 1.02), 0.0794	0.90 (0.72, 1.13), 0.3626
9 ≤ TyG <10	29.02	1.18 (0.96, 1.45), 0.1153	0.84 (0.68, 1.03), 0.0923	0.85 (0.66, 1.10), 0.2127
TyG ≥ 10	43.45	1.78 (1.39, 2.28), <0.0001	1.56 (1.22, 1.99), 0.0005	1.56 (1.14, 2.15), 0.0061
P for trend		<0.001	0.002	0.099
**Cardiovascular mortality**				
**TyG index group**				
TyG <8	6.91	1.0	1.0	1.0
8 ≤ TyG <9	6.09	0.87 (0.60, 1.27), 0.4776	0.62 (0.42, 0.90), 0.0125	0.55 (0.36, 0.82), 0.0039
9 ≤ TyG <10	6.95	0.99 (0.67, 1.46), 0.9543	0.72 (0.49, 1.07), 0.1011	0.53 (0.33, 0.86), 0.0092
TyG ≥ 10	12.95	1.84 (1.17, 2.91), 0.0088	1.69 (1.07, 2.67), 0.0252	1.23 (0.67, 2.25), 0.5044
P for trend		0.002	0.002	0.438

*TyG, triglyceride-glucose; HR, hazard ratio; CI, confidence interval; Q, quintiles.*

*Model I adjust for none.*

*Model II adjust for age, gender, and race.*

*Model III adjust for age, gender, race, smoking, marital status, education level, body mass index, systolic blood pressure, estimated glomerular filtration rate, total cholesterol, high density lipoprotein cholesterol, comorbidities (cardiovascular disease and diabetes), and medicine use (antihypertensive drugs, hypoglycemic agents, lipid-lowering drugs, and antiplatelet drugs)*.

Population with TyG ≥ 10 showed a 56% increased risk of ACM compared with those TyG <8. After an adjustment for confounders, the HRs and CIs for CVM from the three groups (8 ≤ TyG <9, 9 ≤ TyG <10, TyG ≥ 10) were 0.55 (0.36, 0.82), 0.53 (0.33, 0.86), and 1.23 (0.67, 2.25), respectively (*P* for trend = 0.43). After excluding type 2 diabetes mellitus or cardiovascular disease in the baseline, in Supplementary Table 1, we found that when comparing with the lowest group of TyG index (TyG <8), the HRs for ACM from TyG ≥ 10 in the fully adjusted model were 2.05 (1.04, 4.04) (*p* = 0.03). However, it was not associated with any change in the risk of CVM.

**Figure 2:**
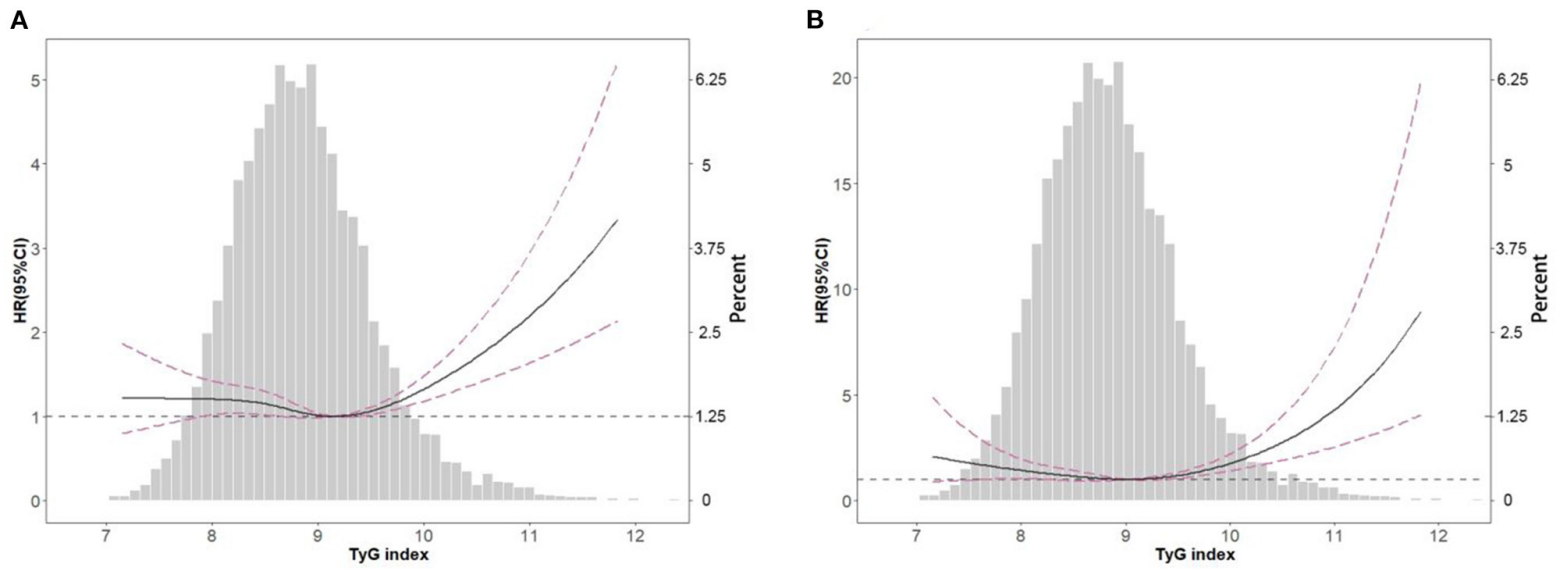
Spline analyses of all-cause **(A)** and cardiovascular **(B)** mortality by TyG index in the overall cohort and the probability distribution histogram is represented in the background (spline analyses were adjusted for age, gender, race, smoking, marital status, education level, body mass index, systolic blood pressure, estimated glomerular filtration rate, total cholesterol, high density lipoprotein cholesterol, cardiovascular disease, diabetes, antihypertensive drugs, hypoglycemic agents, lipid-lowering drugs, and antiplatelet drugs).

In the restricted cubic spline regression models with full adjustment for confounders, the relationships between TyG index, ACM (Figure 2A), and CVM (Figure 2B) were both non-linear in participants with hypertension. The results of two piecewise linear regression model are demonstrated in Table 3. The relationship between TyG index and ACM was non-linear. Subgroup analysis (Table 4) showed non-linear relationship between TyG index and ACM in elderly patients with aged ≥60 years. The cut-off value of TyG for ACM was 9.45. Values higher or lower than 9.45 had more risk of ACM. When TyG index was more than 9.52, the risk for CVM would increase among the whole group.

**Table 3:** The results of two-piecewise linear regression model between triglyceride-glucose index and cause-specific mortality.

	**All-cause mortality**	**Cardiovascular disease mortality**
	**HR (95% CI), *p* value**	**HR (95% CI), *p* value**
Cutoff value	9.45	9.52
< Cut-off value	0.87 (0.76, 0.99), 0.0329	0.81 (0.62, 1.06), 0.1277
≥ Cut-off value	1.73 (1.44, 2.09), <0.0001	2.85 (2.05, 3.98), <0.0001
*p* for log likelihood ratio test	<0.001	<0.001

*TyG, triglyceride-glucose; HR, hazard ratio; CI, confidence interval.*

*The two-piecewise linear regression model were adjusted for age, gender, race, smoking, marital status, education level, body mass index, systolic blood pressure, estimated glomerular filtration rate, total cholesterol, high density lipoprotein cholesterol, comorbidities (cardiovascular disease and diabetes), and medicine use (antihypertensive drugs, hypoglycemic agents, lipid-lowering drugs, and antiplatelet drugs)*.

**Table 4:** Subgroups analysis.

	**N**	**All-cause mortality**		**P for log likelihood ratio test**	**Cardiovascular disease mortality**		**P for log likelihood ratio test**
		**HR (95% CI)**, ***p*** **value**			**HR (95% CI)**, ***p*** **value**		
**Cutoff value, mmol/L**		** <9.45**	**≥9.45**		** <9.52**	**≥9.52**	
**Age**							
≥65	3,442	0.81 (0.70, 0.95), 0.0080	1.48 (1.14, 1.93), 0.0032	<0.001	0.79 (0.58, 1.08), 0.1364	2.36 (1.43, 3.90), 0.0008	0.001
<65	4,402	1.08 (0.83, 1.41), 0.5575	1.59 (1.20, 2.10), 0.0011	0.071	0.86 (0.47, 1.55), 0.6134	2.34 (1.42, 3.87), 0.0009	0.022
**Gender**							
Male	3,889	0.86 (0.72, 1.03), 0.0975	1.78 (1.40, 2.27), <0.0001	<0.001	0.92 (0.65, 1.30), 0.6382	3.18 (2.12, 4.78), <0.0001	<0.001
Female	3,955	0.86 (0.70, 1.05), 0.1439	1.68 (1.25, 2.24), 0.0005	0.001	0.72 (0.46, 1.13), 0.1541	2.23 (1.16, 4.27), 0.0158	0.015
**Race**							
Non-white	3,847	0.83 (0.67, 1.02), 0.0757	1.76 (1.35, 2.28), <0.0001	<0.001	0.71 (0.47, 1.08), 0.1114	3.58 (2.30, 5.57), <0.0001	<0.001
White	3,997	0.91 (0.76, 1.08), 0.2892	1.63 (1.25, 2.13), 0.0003	0.001	0.88 (0.61, 1.26) 0.4722	2.09 (1.21, 3.62), 0.0087	0.020
**Body mass index, kg/m** ^**2**^							
<25	1,683	0.91 (0.71, 1.18), 0.4867	3.08 (1.98, 4.79), <0.0001	<0.001	0.82 (0.49, 1.37), 0.4400	4.72 (2.29, 9.71), <0.0001	<0.001
≥25	6,161	0.85 (0.73, 1.00), 0.0477	1.56 (1.26, 1.92), <0.0001	<0.001	0.79 (0.57, 1.09) 0.1511	2.58 (1.74, 3.80), <0.0001	<0.001

*TyG, triglyceride-glucose; CI, confidence interval.*

*When analyzing a subgroup variable, age, gender, race, smoking, body mass index, systolic blood pressure, estimated glomerular filtration rate, total cholesterol, high density lipoprotein cholesterol, comorbidities (cardiovascular disease, diabetes, and hypertension), and medicine use (antihypertensive drugs, hypoglycemic agents, lipid-lowering drugs, and antiplatelet drugs) were all adjusted except the variable itself*.

**Figure 3:**
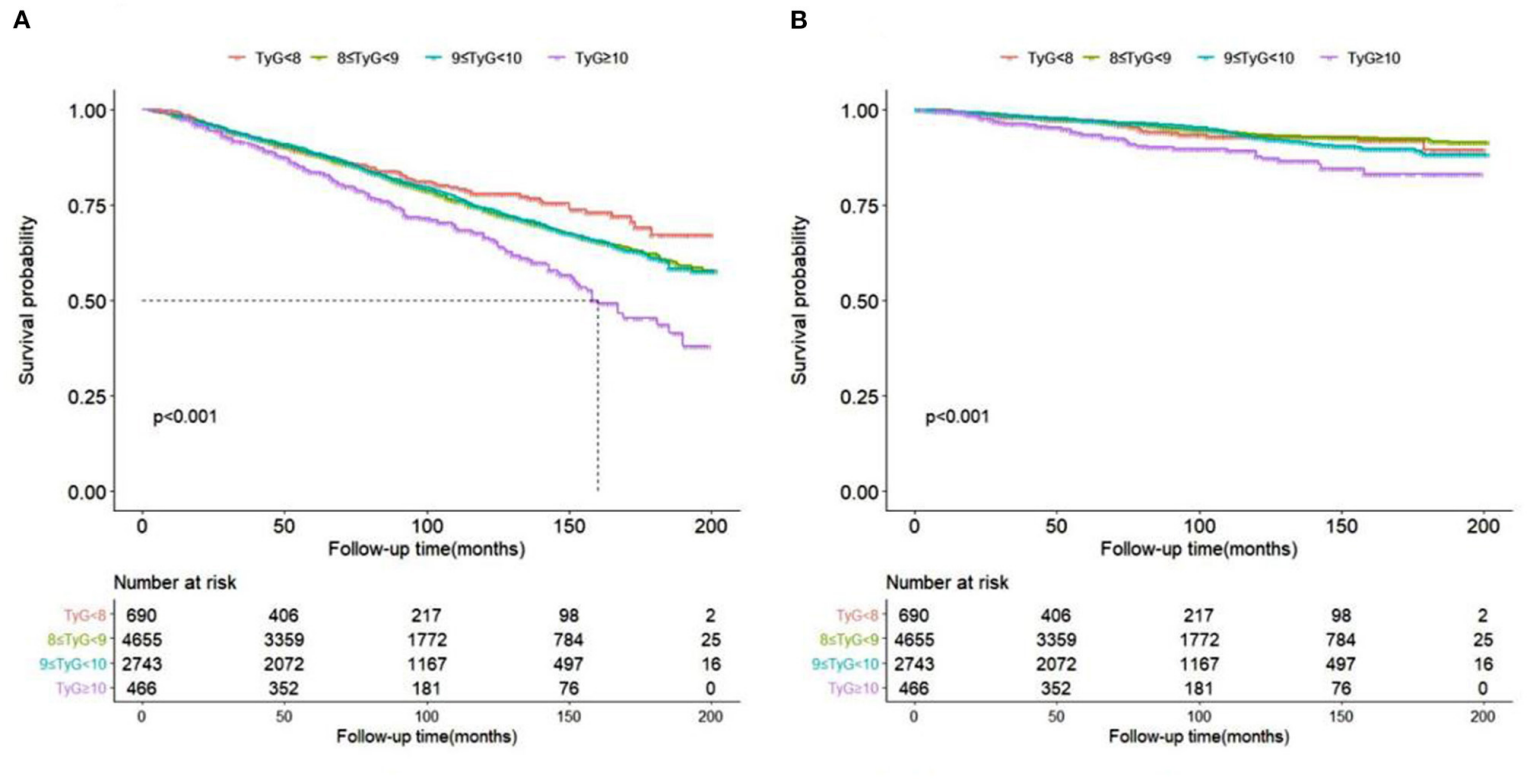
Kaplan-Meier survival curve for all-cause **(A)** and cardiovascular **(B)** mortality by TyG index.

As showed in Kaplan-Meier survival curves (Figure 3), hypertension patients with TyG ≥ 10 had significantly higher ACM (Figure 3A) and CVM (Figure 3B) in the following life (log rank *p* < 0.05).

### Subgroup Analyses

Table 4 has explored the relationship between TyG index, ACM, and CVM as stratified by gender, age, BMI, and race. Only for age ≥ 60 years old patients, the association was significant different between TyG index <9.45 or ≥9.45. When index ≥9.45, risk increased by 48% at every 1 SD increase of TyG index. When index <9.45, risk increased by 19% at every 1 SD decrease of TyG index. Other subgroups only showed a higher TyG index, more than the cut-off value, would present higher risk for ACM and CVM. When TyG index was less than the cut-off value, it showed no difference.

## Discussion

In this retrospective study, we, for the first time, revealed association between IR (TyG index), ACM, and CVM among patients with hypertension in a large cohort study. We demonstrated that the association was non-linear between ACM and TyG index in elderly patient with aged ≥60 years old. But for CVM, only when TyG index was more than 9.52 will the risk for CVM increase among the whole hypertension group.

We also demonstrated the association was non-linear between ACM and TyG index in elderly with aged ≥60 years old. When TyG index ≥9.45, the risk of ACM increased by 48% at every 1 SD increase of TyG index. In contrast, for TyG index <9.45, the risk of ACM increased by 19% as the TyG index decreased by 1 SD. TyG index, a composite indicator based on TG level and FBG value, was shown to be used as a surrogate marker of IR (9, 11, 12). Previous studies probed the relationship between IR and ACM in different population. An observational prospective cohort study that enrolled 15,773 patients with type 2 diabetes showed that highest IR predicts ACM in type 2 diabetes (13). High IR, as measured by HOMA-IR, identified postmenopausal women at higher risk for cancer-specific and ACM (14). In addition, A meta-analysis included seven articles involving 26,976 non-diabetic adults showed that the highest HOMA-IR increased the risk of ACM by 34% when compared with the lowest category (15). For elderly people, there were different conclusions. Among community-dwelling older individuals over 65 years, HOMA-IR and low-grade systemic inflammation was associated with a 9-year ACM and CVM risk (16). Aging and high C-reactive protein levels were, usually, risk factors for cardiovascular disease mortality. We did not evaluate systemic inflammation in our study. A community-based prospective study selected participants with age ≥65 years in Korea (17). Elderly subjects in the fifth and first quintile of HOMA-IR values had increased rates of ACM and CVM as there is a threshold level of HOMA-IR in relation to mortality. The HOMA-IR was lower in Korea study (1st quintile ≤ 0.67 and 5th quintile > 1.50). Our work showed similar with the study in Korea; however, we used the TyG index to evaluate IR, not HOMA-IR, in elderly hypertension patients.

A previous study showed that there was a striking U-shaped relationship between FBG levels and in-hospital and 3-year mortality in older patients with acute myocardial infarction (AMI) (18). The study observed that mild to moderately low FBG levels ( ≤ 5 mmol/L, 90 mg/dl) were associated with a relative increase in mortality risk. Hypoglycemia and rapid changes in blood glucose level were shown to increase levels of counterregulatory hormones, such as epinephrine and norepinephrine, which may induce vasoconstriction and platelet aggregation, resulting in ischemia of cardiovascular or cerebrovascular (19). In the elderly population, the decrease in skeletal muscle mass is generally accompanied by weight loss. A study that recruited elderly individuals (≥65 years) in Taiwan showed that elderly subjects with sarcopenic obesity had the highest ACM (19). In contrast, except for the TG level, none of the other lipid profile indices were related to ACM in patients aged over 75 years (20). The mortality risk decreased by 17% for each 1 mmol/L increase serum level of TG in ZODIAC study (20). The lower TG had higher risk of ACM for elderly population. One study, which enrolled individuals aged 65 and older residing in northern Manhattan in 1992–1994 and 1999–2002, showed low cholesterol level was a robust predictor of mortality in the non-demented elderly and may be a surrogate of frailty or subclinical disease (21). Our study was collected from 1999 to 2014 in the same country as the study (21), so the life diet structure and level were similar. The mean TG of the patients with mortality was 155 mg/dl (21). Therefore, the finding of our study, specifically of the 1 SD decrease of TyG index when TyG index <9.45 in elderly with aged ≥60 years and 19% increased risk of ACM was persuasive. Other factors, such as body composition and nutritional state, might be predictors of mortality in the elderly population (17). Older age, anemia, lower baseline hemoglobin level (22), and lower BMI had a greater effect on mortality.

Only when TyG index ≥9.52, TyG-index showed significant with CVM. A previous study had reported that IR was associated with cardiovascular disease (23). The relationship between IR and endothelial dysfunction may the underlying biological mechanism of cardiovascular. This mostly includes inflammation (24) and functional impairment in the endothelium of blood vessels (25) which leads to atherosclerosis and cardiovascular disease. This simple, convenient, and low-cost TyG index was of research interest in many Countries and could be used to screen for IR in the hypertensive community. Furthermore, Park et al. (26) reported that TyG-index is an independent predictor of coronary artery calcium progression. We first investigate relationship between TyG index and CVM and ACM in hypertension. The Kaplan-Meier survival curve by TyG index groups also confirmed that the higher TyG index was associated with increased higher incident of ACM and CVM.

Our study still had several limitations that need to be mentioned. First, we did not have multiple-time monitoring of the TyG index along the follow-up, which may provide more information. Secondly, we did not compare the TyG index with HOMA-IR and the hyperinsulinemic-euglycemic clamp test. Thirdly, nutritional habits or energy intake were not recorded. Although we did not adjust for these potential confounding variables, we used other variables, such as BMI or cholesterol levels, which are indirectly related to nutritional habits or energy intake. Finally, this was a population-based study conducted among hypertension participants in the United States. Therefore, our findings may not be generalizable to other populations.

## Conclusion

We first time revealed association between IR (TyG index), ACM, and CVM among patients with hypertension. We demonstrated that the association was non-linear between ACM and TyG index in elderly with aged ≥60 years. But for CVM, higher TyG index had high risk among patients with hypertension only when TyG index ≥9.52.

## Data Availability Statement

The datasets presented in this study can be found in online repositories. The names of the repository/repositories and accession number(s) can be found in the article/Supplementary Material.

## Ethics Statement

The studies involving human participants were reviewed and approved by the Institutional Review Board of the Centers for Disease Control and Prevention. The patients/participants provided their written informed consent to participate in this study.

## Author Contributions

DZ and Y-qF contributed to the conception or design of the study and drafted the manuscript. X-cL contributed to the acquisition of data, interpretation of data, and analysis of data. Y-qH and LK contributed to the interpretation of data and critical revision of the article for important intellectual content. All authors gave final approval of the article.

## Funding

This research was supported by Science and Technology Plan Program of Guangzhou (201803040012), the Key Area R&D Program of Guangdong Province (No. 2019B020227005), Guangdong Provincial People's Hospital Clinical Research Fund (Y012018085), the Fundamental and Applied Basic Research Foundation Project of Guangdong Province (2020A1515010738), High-level Hospital Construction Project of Guangdong Provincial People's Hospital (DFJH2020022), and Guangdong Provincial Clinical Research Center for Cardiovascular Disease (2020B1111170011).

## Conflict of Interest

The authors declare that the research was conducted in the absence of any commercial or financial relationships that could be construed as a potential conflict of interest.

## Publisher's Note

All claims expressed in this article are solely those of the authors and do not necessarily represent those of their affiliated organizations, or those of the publisher, the editors and the reviewers. Any product that may be evaluated in this article, or claim that may be made by its manufacturer, is not guaranteed or endorsed by the publisher.

## Abbreviations

IR: insulin resistance
CVM: cardiovascular mortality
ACM: all-cause mortality
TyG index: triglyceride glycemic index
HOMA-IR: homeostatic model assessment IR
SBP: systolic blood pressure
DBP: diastolic blood pressure
TC: total cholesterol
TG: triglyceride
LDL-C: low-density lipoprotein cholesterol
HDL-C: high-density lipoprotein cholesterol
FBG: fasting blood glucose
eGFR: estimated glomerular filtration rate
BMI: body mass index
HbA1C: hemoglobin A1c.
