# Supplementary material for: A Non-Linear Association of Triglyceride Glycemic Index With Cardiovascular and All-Cause Mortality Among Patients With Hypertension
Source: Front Cardiovasc Med. 2022 Jan 27;8:778038. doi: 10.3389/fcvm.2021.778038 (PMC8828937; doi:10.3389/fcvm.2021.778038)
Supplement: Supplementary file 1 [file Data_Sheet_1.docx]

Table S1 Multivariate Cox regression analysis of TyG index with cause-specific mortality in patients without T2DM AND CVD

|  | Case/Number | Event rate/ 1000 person-years | Model I  HR (95%CI), p-value | Model II  HR (95%CI), p-value | Model III  HR (95%CI), p-value |
| --- | --- | --- | --- | --- | --- |
| All-cause mortality | | | | | |
| TyG index group |  |  |  |  |  |
| TyG<8 | 60/511 | 17.60 | 1.0 | 1.0 | 1.0 |
| 8≤TyG<9 | 530/3075 | 21.82 | 1.20 (0.92, 1.56) 0.1880 | 0.79 (0.61, 1.04) 0.0878 | 0.93 (0.68, 1.26) 0.6349 |
| 9≤TyG<10 | 215/1277 | 19.74 | 1.07 (0.80, 1.42) 0.6637 | 0.77 (0.58, 1.03) 0.0734 | 1.01 (0.71, 1.44) 0.9518 |
| TyG≥10 | 13/81 | 17.97 | 0.97 (0.53, 1.77) 0.9185 | 1.05 (0.58, 1.92) 0.8627 | 2.05 (1.04, 4.04) 0.0378 |
| P for trend |  |  | 0.5334 | 0.3702 | 0.2494 |
| Cardiovascular mortality | | | | | |
| TyG index group |  |  |  |  |  |
| TyG<8 | 14/511 | 4.11 | 1.0 | 1.0 | 1.0 |
| 8≤TyG<9 | 94/3075 | 3.87 | 0.94 (0.54, 1.65) 0.8318 | 0.63 (0.36, 1.12) 0.1145 | 0.63 (0.34, 1.19) 0.1555 |
| 9≤TyG<10 | 40/1277 | 3.67 | 0.89 (0.49, 1.64) 0.7138 | 0.70 (0.38, 1.28) 0.2455 | 0.63 (0.29, 1.35) 0.2329 |
| TyG≥10 | 2/81 | 2.77 | 0.66 (0.15, 2.90) 0.5798 | 0.89 (0.20, 3.93) 0.8774 | 1.44 (0.29, 7.21) 0.6604 |
| P for trend |  |  | 0.5795 | 0.7219 | 0.6284 |

TyG, Triglyceride-glucose; HR, hazard ratio; CI, confidence interval; Q: quintiles; T2DM: Type 2 diabetes mellitus; CVD: Cardiovascular disease

Model I adjust for none

Model II adjust for age, gender, and race

Model III adjust for age, gender, race, smoking, marital status, education level, body mass index, systolic blood pressure, estimated glomerular filtration rate, total cholesterol, high density lipoprotein cholesterol, and medicine use (antihypertensive drugs, and lipid-lowering drugs).
